# Asymmetric scapuloperoneal phenotype of *MATR3*-related distal myopathy: case series

**DOI:** 10.3389/fgene.2024.1414928

**Published:** 2024-08-13

**Authors:** Aysylu Murtazina, Dmitrii Subbotin, Anna Kuchina, Olga Gilvanova, Daniil Degterev, Olga Shchagina, Tatiana Cherevatova, Maria Bulakh, Darya Sherstyukova, Oksana Ryzhkova, Olga Kurushina, Mikhail Skoblov, Artem Borovikov, Sergey Kutsev

**Affiliations:** ^1^ Research Centre for Medical Genetics, Moscow, Russia; ^2^ Loginov Moscow Clinical Scientific Center, Moscow, Russia; ^3^ Department of Neurology, Neurosurgery, Medical Genetics, Volgograd State Medical University, Volgograd, Russia

**Keywords:** MATR3, distal myopathy, scapuloperoneal phenotype, phenotypic variability, FSHD, ALS

## Abstract

Recent research has sparked a discussion on the spectrum of diseases linked to the *MATR3* gene associated with amyotrophic lateral sclerosis and distal myopathy with vocal cord and pharyngeal weakness (VCPDM). To date, fewer than 50 cases of VCPDM have been reported in the literature. We aim to build upon the work of previous researchers by gathering additional information about VCPDM. In this study, we present six patients from four unrelated families affected by VCPDM. Our observations include patients exhibiting both the typical phenotype associated with MATR3-related distal myopathy and rare symptomatic manifestations of the disease. Notably, two cases presented with an asymmetric scapuloperoneal phenotype, leading in one case to an initial misdiagnosis of facioscapulohumeral muscular dystrophy.

## 1 Introduction

Distal myopathies are a group of hereditary primary muscle disorders that are characterized by extensive phenotypic and genetic variability. Currently, more than 25 genes associated with this nosology have been identified (Milone and Liewluck, 2019; Savarese et al., 2020). Heterozygous variants in the *MATR3* gene cause amyotrophic lateral sclerosis (ALS) type 21 and distal myopathy with vocal cord and pharyngeal weakness (VCPDM) inherited in autosomal dominant manner (Senderek et al., 2009; Johnson et al., 2014).

The *MATR3* gene encodes matrin-3, a highly conserved nuclear matrix protein that binds and interacts with nucleic acids (Belgrader et al., 1991; Hibino et al., 2006). It plays a role in RNA transcription and processing, as well as mRNA stabilization (Hibino et al., 2006). Structurally, the protein includes two zinc finger domains that interact with DNA, along with two domains that bind RNA (Belgrader et al., 1991; Hibino et al., 2006; Salton et al., 2011; Johnson et al., 2014).

The association of the 5q31 loсus with VCPDM was initially reported in 1998 (Feit et al., 1998). Almost a decade later, this disease was associated with a pathogenic missense variant, p.S85C, in the *MATR3* gene across all patients (Senderek et al., 2009). The central features of the clinical presentation in the reported cases included muscle weakness and atrophy, primarily affecting the distal extremities, with a pronounced impact on the anterior compartment of lower leg muscles. Additionally, patients exhibited dysphonia and dysphagia. Onset of the disease was uniformly observed in adulthood, ranging from 35 to 57 years, and creatine kinase values varied from within the normal range to 8 times higher than normal (Feit et al., 1998; Senderek et al., 2009). Later, the authors reclassified this condition as slowly progressive ALS based on the signs of upper motor neuron involvement in some patients. This occurrence sparked further exploration into the association familial ALS with the *MATR3* gene. Subsequently, in cases of clinically definite ALS other causative nucleotide variants in the *MATR3* gene were identified (Johnson et al., 2014).

Currently, only one single nucleotide variant in the *MATR3* gene NM_018834.6:c.254C>G, p.S85C, has been identified in all reported cases of VCPDM. Despite the identification of a common clinical core, the manifestations of the disease vary significantly among patients, both in terms of the age of onset and the severity of symptoms. The absence of pathognomonic clinical signs, coupled with the high genetic heterogeneity of distal myopathies, complicates the process of a differential diagnosis.

In this study, we present a case series of distal myopathy associated with the heterozygous previously reported p.S85C variant in the *MATR3* gene.

## 2 Subjects and methods

The clinical and genetic characteristics of six patients from four unrelated families were investigated, comprising three male and three female patients. The age of the patients at the time of examination ranged from 36 to 65 years. All patients underwent neurophysiological evaluation, while four patients also underwent lower limb muscle magnetic resonance imaging (MRI) and spirometry testing.

The clinical examination also involved assessing dysphagia through a water swallowing test. Participants were instructed to drink 100 mL of water as quickly as possible without interruption. The duration from onset of swallowing to completing the drinking task was recorded in seconds, and the speed of swallowing (mL/s) was determined by dividing 100 mL by the time taken to drink. A swallowing speed exceeding 10 mL/s was regarded as within the normal range.

A spirometry testing was conducted in the sitting and supine position. Maximum vital capacity and forced vital capacity as percentage of predicted value, as well as the difference in the forced vital capacity in both positions were assessed in three patients. Electrocardiography and cardiac ultrasound were performed on six and three patients, respectively. Neurophysiological examination (Neuro-MVP-micro, Neurosoft, Ivanovo, Russia) included nerve conduction study of peripheral nerves and needle electromyography of proximal and distal limb muscles (four patients), only distal muscles of upper and lower limbs (one patient), proximal and distal lower limb muscles (one patient).

Lower limb muscle MRI (axial T1-weighted and axial T2-weighted images) was conducted for four patients using a 3T magnetic resonance scanner (Siemens MAGNETOM Skyra, Erlangen, Germany). Increased signal intensity on the T1-weighted images was semi quantitatively assessed using the four-point Mercuri scale, which reflects stages of muscle fatty infiltration (Mercuri et al., 2007).

For molecular genetic studies, blood samples were collected from the probands, affected and healthy family members. Genomic DNA was extracted using standard methods. An exome sequencing was performed for all probands. Target enrichment was performed using Illumina TruSeq^®^ ExomeKit (Illumina, San Diego, CA, United States), and custom oligonucleotides (IDT xGen Exome Research Panel v1.0) included coding regions of over 20,000 protein-coding genes. Paired-end sequencing (2 × 150 bp) was carried out on an Illumina NextSeq 500. The sequencing data were processed using Illumina’s Basespace software (Enrichment 3.1.0). Variant filtering was based on their frequency, with variants having a frequency of less than 1% in The Genome Aggregation Database (gnomAD v.2.1.1) and coding region sequence effects such as missense, nonsense, coding indels, and splice sites being considered. The clinical significance of the variants was evaluated using ACMG criteria for variant interpretation (Richards et al., 2015). To validate the pathogenic variant in the proband and affected family members, Sanger sequencing was performed using the Applied Biosystems 3,130 xl Genetic Analyzer (HITACHI, Applied Biosystems Group of The Applera Corporation Japan, Waltham, MA, United States).

One patient underwent a PCR-based approach to detect D4Z4 repeat contraction (Zernov et al., 2021). The genomic DNA extracted from peripheral blood lymphocytes was embedded in agarose plugs. A portion of the plug, which had been pre-treated with the EcoRI restriction enzyme, was used for pulsed-field gel electrophoresis. The gel lines of the samples were then fragmented based on the lengths of the ladder bands. Each gel fragment was completely melted and diluted fivefold with PCR-grade water, and the resulting solutions were directly used for qPCR. For each gel fragment derived from a single patient, two separate qPCR analyses were conducted using specific primers to identify the contracted allele and the presence of a permissive haplotype on this allele. The qPCR data were analyzed using the 2^−ΔΔCT^ method.

Patients and their relatives provided written informed consent for the publication of the data.

## 3 Case descriptions

**FIGURE 1:**
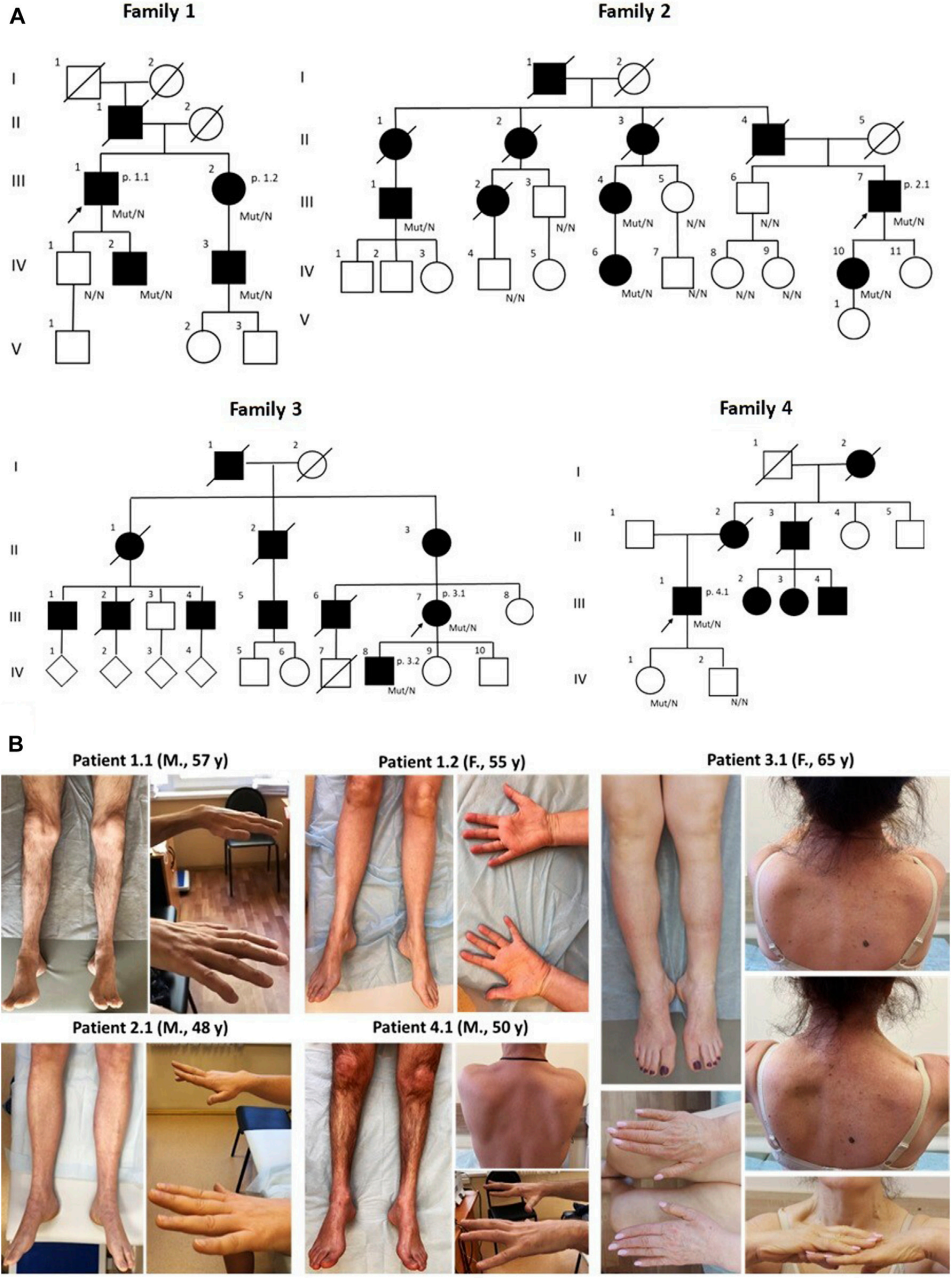
The pedigrees of all patients correspond to the autosomal dominant type of inheritance. Diamond symbols indicate offspring, whose gender is unknown **(A)** Clinical presentations of five patients with VCPDM **(B)** The illustrations represent typical distal muscle atrophy observed in all patients. Note scapular winging and a clear asymmetric pattern of muscle involvement in two patients 3.1 and 4.1. M, male; F, female; y, years.

The study included six patients (three male, three female) from four families, aged 36–65 years (Table 1). Notably, three unrelated families were ethnic Tatars. Pedigrees are characterized by a significant number of affected family members consistent with autosomal dominant manner of inheritance (Figure 1). The missense variant p.S85C in the *MATR3* gene was detected in all of the probands by exome sequencing.

**TABLE 1:** Clinical phenotype of patients with VCPDM. VC, vital capacity, percentage of predicted value; FVC sit., forced vital capacity in sitting position; FVC sup., forced vital capacity in supine position; FVC diff., forced vital capacity, the difference in the sitting and supine position; CK, Creatine kinase; EMG, electromyography; VL, vastus lateralis; TA, tibialis anterior; Delt., deltoideus; FDI, first dorsal interosseous; FP, fibrillation potentials; PSW, positive sharp waves; SA, spontaneous activity; CRD, complex repetitive discharges; ECG, electrocardiography; y, years; DD, disease duration; m, male; f, female; n/d, no data; r., reflex.

Patient	1.1	1.2	2.1	3.1	3.2	4.1
Age (y)/gender	57/m	55/f	48/m	65/f	36/m	50/m
Age of onset (y)	40	49	40	30	34	40
DD (y)	17	6	8	35	2	10
First symptoms	gait disturbances	gait disturbances	foot drop	gait disturbances	gait disturbances	foot drop
Pharyngeal reflex	brisk	normal	normal	D > S	brisk	normal
Dysphonia/dysphagia	yes	yes/no	yes	no/yes	no	no/yes
Upper limb weakness (MRC)	finger extensors (4/5), hand muscles (4/5)	forearm muscles (3/5), hand muscles (3/5)	finger extensors (3/5), hand muscles (1/5)	deltoid bilaterally (4/5), forearm right (3/5) and left (4/5), hand muscles (3/5)	hand muscles (4/5)	forearm muscles (4/5), hand muscles (0-3/5)
Lower limb weakness (MRC)	lower leg muscles (3/5), big toe extensors (1/5), foot muscles (4/5)	lower leg muscles (4/5), big toe extensors (2/5), foot muscles (3/5)	pelvic girdle (4/5), hamstrings (4/5), foot extensors (0/5), foot flexors (4/5), foot muscles (1/5)	pelvic girdle (4/5), hamstrings (4/5), lower leg muscles (0-1/5)	big toe extensors (4/5)	lower leg muscles (3-4/5), foot muscles (2-3/5)
Gait disturbance	steppage	steppage	steppage and waddling gait	steppage and waddling gait	no	steppage
Hand tremor	Yes	No	yes	no	no	no
Arm reflexes	brachioradialis r. reduced	brisk	brachioradialis r. reduced	triceps and left biceps r. reduced, brachioradialis r. absent	brachioradialis r. reduced	brachioradialis r. absent
Leg reflexes	ankle r. absent	ankle r. absent	ankle r. absent	ankle r. absent	patellar and ankle r. absent	brisk patellar r., ankle r. absent
Additional signs	No	pyramidal signs in hands, impaired foot vibration sensitivity	mild facial weakness, lumbar hyperlordosis	asymmetric abdominal protrusion, lumbar hyperlordosis, scapular winging (S > D), ptosis (D > S)	no	left side hearing impairment, scapular winging (D > S)
FVC sit. (%)	n/d	63	47	107	n/d	66
FVC sup. (%)	n/d	n/d	36	88	n/d	43
FVC diff. (%)	n/d	n/d	24	19	n/d	23
CK (U/L)	603	236	604	240	480	969
Needle EMG	VL, TA: myogenic, FP (+2), PSW (+2)	Delt., FDI, VL, TA: myogenic, no SA	EDC and TA: myogenic, no SA	Delt., FDI, VL, TA: myogenic, FP (+1), PSW (+1), CRD	EDC, VL, TA: myogenic, FP (+2), PSW (+2)	Delt., FDI, VL, TA: myogenic, no SA
ECG	normal	incomplete right bundle branch block	normal	nonspecific ventricular repolarization abnormalities	normal	normal
Cardiac ultrasound	n/d	normal	n/d	mild left ventricular diastolic dysfunction, all valves mild regurgitation	n/d	mild mitral, pulmonary and tricuspid valves regurgitation

In all patients, the disease manifested between the ages of 30 and 49 years, with initial symptoms of weakness in the distal leg muscles and gait disturbances. None of the patients exhibited evidence of delayed motor development nor reported difficulties in performing physical exercises during childhood or adolescence. Additionally, younger family members below 30 years old, who carried the pathogenic variant, did not display any obvious symptoms of the disease.

Clinical examination revealed selective involvement of distal limb muscles in all patients. Notably, weakness of the big toe extensors was more pronounced (0-2/5 on the Medical Research Council scale (MRC)) in five patients, with the exception of patient 3.2, while other muscles in the lower leg and feet were moderately affected. Two individuals exhibited proximal leg weakness, reaching up to 4/5 on the MRC scale, leading to a waddling gait. Steppage gait and/or difficulties walking on heels and toes were common signs observed in all patients. Additionally, varying degrees of weakness in hand and forearm muscles were observed in five patients. Special attention was drawn to the selectivity of the hand finger extensor weakness, particularly pronounced in patients 2.1, 3.1, and 4.1 (Figure 1). An interesting finding is the asymmetric atrophy of the left extensor digitorum brevis in patient 3.2. In other patients, although there was some atrophy of the muscles in the feet, the extensor digitorum brevis maintained contour well under tension and was not weak.

In two patients (3.1 and 4.1), we observed weakness of the proximal arm muscles (patient 3.1) and asymmetric scapular winging that in combination with peroneal weakness led us initially to the diagnosis of scapuloperoneal myopathy. One of these individuals, a 65-year-old female patient (3.1), had clear features associated with facioscapulohumeral muscular dystrophy (FSHD). Alongside asymmetric shoulder girdle involvement and scapular winging, she exhibited abdominal protrusion, lumbar hyperlordosis and specific combination of steppage and waddling gait. Due to these clinical features, the patient initially underwent a PCR-based approach to detect D4Z4 repeat contraction, which revealed 12 D4Z4 repeats. Subsequently, whole exome sequencing was conducted to identify causative variants in genes associated with FSHD type 2. However, no variants were detected in these genes, including the *SMCHD1* gene.

Ankle reflexes were absent in all patients, while arm reflexes were reduced in four patients. Notably, patient 1.2 exhibited a unique neurological status characterized by brisk tendon reflexes in the hands and the presence of pyramidal signs in the lower limbs at the age of 55, which could be explained by the combination of myopathy and ALS features in one individual. Hand tremor was noticed in patients 1.1 and 2.1.

Bulbar signs were observed in five patients. Dysphagia was detected in four patients, while dysphonia was reported in three cases. In two cases (patients 2.1 and 4.1), dysphagia or dysphonia developed after 8 years from the onset of the disease, and in another case, only after 31 years (patient 3.1). Notably, patient 1.2 had a hoarse voice. None of the cases exhibited atrophy or fasciculation of the tongue muscles. To assess dysphagia, the swallowing test was performed in three patients, revealing a decreased swallowing speed in all examined patients, ranging from 3.6 to 8.3 mL/s (normal reference is more than 10 mL/s).

Two patients (patients 1.2 and 2.1) reported moderate respiratory difficulties in the supine position at night. A spirometry was conducted on three patients (patients 2.1, 3.1 and 4.1), revealing abnormal values in all cases. Vital capacity was lower than 75% of the reference value in two patients (patients 2.1 and 4.1). All three patients exhibited more than a 15% decrease in forced vital capacity while in the supine position, indicating diaphragmatic weakness.

The serum creatine kinase level ranged from slightly elevated to three times higher than normal values in all patients. In all cases, needle electromyography of limb muscles revealed myogenic changes. Pathological spontaneous activity was observed, characterized as mild in one patient, moderate in two patients, and manifested as fibrillation potentials and positive sharp waves. Additionally, complex repetitive discharge was identified in one patient.

According to the electrocardiographic data, patients 1.2 and 3.1 exhibited signs of impaired cardiac conduction (Table 1). Cardiac ultrasound revealed mild changes also in two patients (3.1 and 4.1).

**FIGURE 2:**
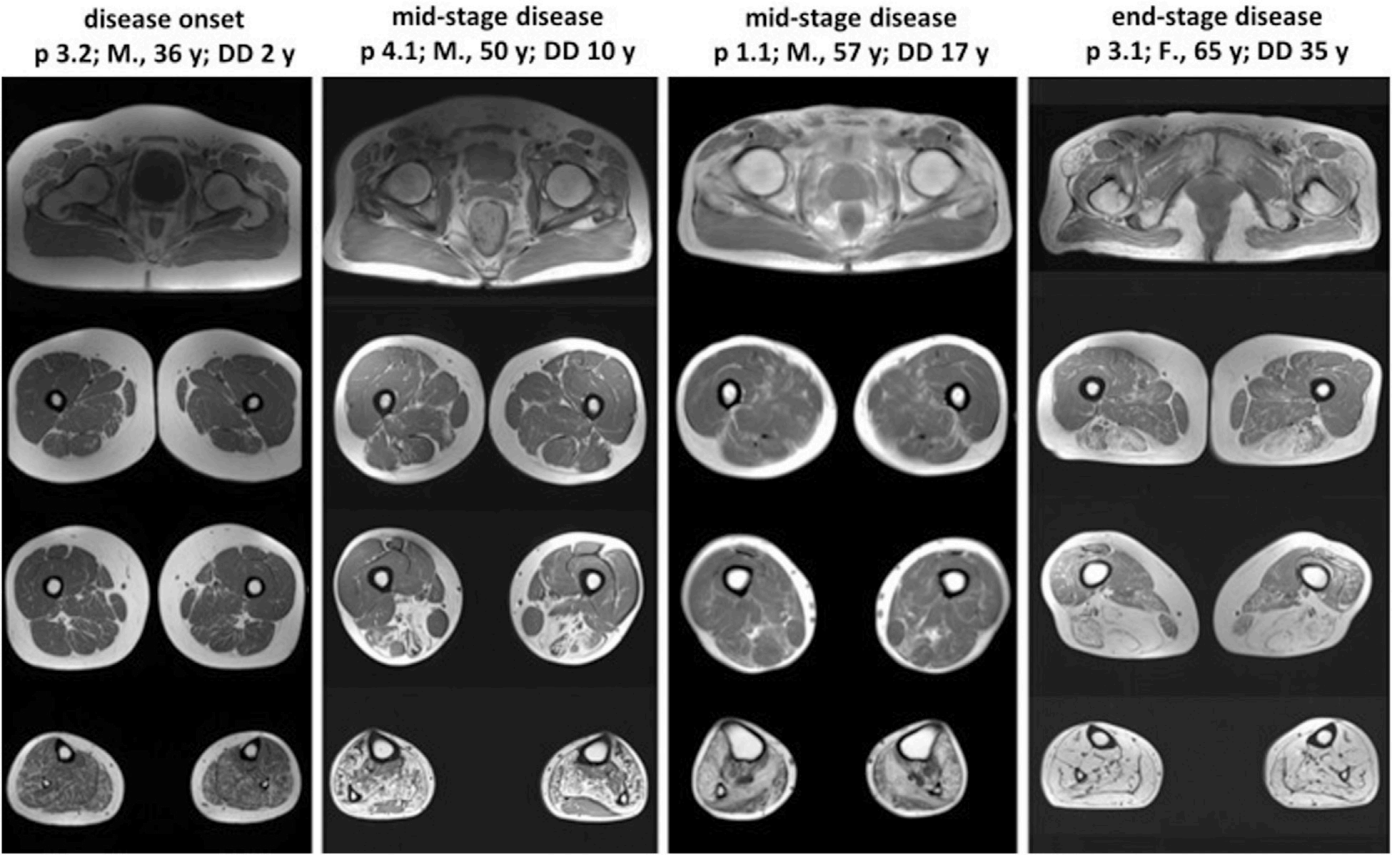
Lower limb muscle MRI in patients with different stages of VCPDM. The characteristic pattern of muscle involvement was observed in patients with the mid-stage of the disease. T1-weighted MRI revealed the typical involvement of lower leg muscles, with relatively spared tibialis posterior and lateral gastrocnemius. The patient with disease onset demonstrated mild diffusely affected posterior compartment of lower leg muscles. In the patient with end-stage disease, complete fatty replacement of lower leg muscles was noted. The most pronounced affected thigh muscles were the long head of the biceps femoris and the semimembranosus with spared gracilis in all patients. M, male; F, female; y, years; p, patient; DD, disease duration.

Lower limb muscle MRI was performed on patients 1.1, 3.1, 3.2, and 4.1, each exhibiting varying disease durations (Figure 2). Across all patients, the lower leg muscles showed more extensive involvement compared to the thigh muscles. The pattern and severity of muscle involvement on T1-weighted images correlated with the duration of the disease. Muscle fatty replacement was symmetric in all patients, including patient 3.1, who exhibited clearly asymmetric clinical features. Patients in the mid-stage of the disease (1.1 and 4.1) exhibited a characteristic muscle involvement pattern, with the posterior tibial and lateral gastrocnemius muscles relatively spared. Patient 3.2, in the early disease stage, showed diffuse, slight involvement of lower leg muscles, except for the tibialis anterior and tibialis posterior muscles. Conversely, patient 3.1, in the advanced disease stage, demonstrated total fatty replacement of lower leg muscles. In the thigh, muscles of the posterior compartment, particularly the long head of the biceps femoris and the semimembranosus muscles, were most affected.

Due to the lack of specific therapy, all patients were given only general recommendations for physiotherapy, correction of swallowing disorders, and dynamic monitoring of respiratory functions. One patient began to regularly engage in aerobic exercises and noticed an improvement in her condition, with a subjective increase in muscle strength over 1 year of follow-up.

## 4 Discussion

Here, we present a series of six patients with VCPDM, expanding the worldwide cohort of patients (Feit et al., 1998; Senderek et al., 2009; Müller et al., 2014; Palmio et al., 2016; Barp et al., 2018). In all our patients, the disease is caused by a recurrent pathogenic missense variant p.S85C in the *MATR3* gene, as observed in all previously reported cases of this myopathy.

Important functions of MATR3 include interacting with other nuclear matrix proteins to form the internal fibrogranular network and retaining defective RNAs within the nucleus, among others (Iradi et al., 2018). MATR3 is also believed to play a role in gene regulatory functions, including DNA replication and transcription (Cha et al., 2021). In previous studies on cultured cells and *Drosophila* models, no significant difference was found in the effects specific to the distal myopathy variant p.S85C and the missense variants associated with ALS (Iradi et al., 2018; Ramesh et al., 2020). Given that missense variants have been described for both diseases, it can be assumed that the amino acid substitution at position 85 leads to a gain-of-function event, which is associated with the dysregulation of genes expressed in skeletal muscles.

All cases in our study exhibited a typical disease onset after the age of 30, characterized by weakness of distal leg muscles. The characteristic initial symptoms of VCPDM include the selective involvement of foot extensors, resulting in gait disturbances, foot drop, and frequent falls, observed in 80% of patients as well as in all our cases (Table 2). Hand muscle weakness is also an early sign observed in all our patients, including patient 3.2, at the disease onset stage.

**TABLE 2:** The representation of clinical features in previously reported and current study.

Clinical features	Current study	Previously reported cases	All cases
Onset ≥30 years	6/6	48/48	54/54 (100%)
Initial involvement of distal lower limb muscles	6/6	33/43	39/49 (80%)
Initial involvement of distal upper limb muscles	0/6	5/43	5/49 (10%)
Initial involvement of proximal lower limb muscles	0/6	5/20	5/26 (19%)
Initial voice pathology, dysphagia and/or dysarthria	0/6	4/48	4/54 (7%)
Distal lower limb muscles weakness	6/6	43/45	49/51 (96%)
Distal upper limb muscles weakness	6/6	42/49	48/55 (87%)
Proximal lower limb muscles weakness	4/6	17/21	21/27 (78%)
Proximal upper limb muscles weakness	1/6	22/44	23/50 (46%)
Scapular winging	2/6	1/17	3/23 (13%)
Facial weakness	1/6	0/17	1/23 (4%)
Ptosis	1/6	1/17	2/23 (7%)
Neck muscle weakness	0/6	4/19	4/24 (17%)
Voice pathology, dysphagia and/or dysarthria	5/6	28/44	33/50 (66%)
Respiratory impairment	3/3	11/34	14/37 (38%)
Restriction of ambulation	0/6	3/21	3/27 (11%)
Myalgia	0/6	5/17	5/23 (21%)
Creatine kinase, ≥ 2-fold	3/5	23/42	26/47 (55%)

Five patients exhibited dysphagia and/or dysphonia, often without actively paying attention to it. One patient reported a long-term hoarse voice that had never been associated with the disease. Therefore, it is crucial to conduct an objective examination of swallowing and respiratory function. In all three patients for whom spirometry was performed, an abnormal forced vital capacity difference between sitting and supine positions was detected. Two patients complained of respiratory difficulties at night.

Some patients with the recurrent variant p.S85C may exhibit additional features of pyramidal involvement (Palmio et al., 2016; Cavalli et al., 2021). In our cohort, we observed one patient who presented with pyramidal signs in her hands at the age of 55 years. However, this patient was diagnosed with distal myopathy, which was confirmed by clear muscle MRI and needle electromyography results. To date, there is no definitive explanation for why some patients present with a combination of ALS and myopathy symptoms, while others manifest only an isolated phenotype (Johnson et al., 2014).

Our study included patients with varying disease durations, enabling us to compare muscle fatty replacement at different stages of the disease. The MRI pattern of muscle involvement in our patients is consistent with previously reported cases (Müller et al., 2014; Palmio et al., 2016; Barp et al., 2018; Mensch et al., 2020). Patients in the mid-stage of the disease exhibited relatively spared tibialis posterior and lateral gastrocnemius muscles at the lower leg level, which are characteristic features of VCPDM (Mensch et al., 2020). It is noteworthy that gracilis was consistently the least affected muscle in all patients, including those in the end-stage of the disease. Despite asymmetric clinical features observed in two individuals (patients 3.1 and 4.1), all patients exhibited symmetric muscle fatty replacement. This observation aligns with findings from previous studies investigating the MRI patterns in patients with VCPDM (Barp et al., 2018; Mensch et al., 2020).

Two out of six patients demonstrated features of asymmetric scapuloperoneal myopathy, which, in one case, necessitated the exclusion of FSHD through targeted genetic molecular analysis. Notably, in this proband, 12 macrosatellite D4Z4 repeats were detected, falling within the lower border of the normal range. Whole exome sequencing did not reveal any causative variants in the genes associated with FSHD type 2. Scapular winging has been rarely described in some patients previously (Müller et al., 2014; Barp et al., 2018). Several other reports have mentioned asymmetry in the early stages of the disease (Feit et al., 1998; Müller et al., 2014; Barp et al., 2018). The clinical overlap between VCPDM and FSHD is an intriguing issue for further investigation. A recent study by Runfola et al. demonstrated that MATR3 serves as a cellular factor regulating the expression and activity of the transcription factor double homeobox 4 (DUX4) (Runfola et al., 2023). It is assumed that DUX4 activates a proapoptotic transcription program, inhibiting myogenic differentiation and leading to FSHD (Mocciaro et al., 2021).

Generally, the differential diagnosis between VCPDM and FSHD is not complicated. VCPDM typically presents with a distal phenotype accompanied by some bulbar disturbances. In contrast, FSHD manifests with facial and shoulder girdle weakness. However, some individuals with VCPDM may develop facial muscle weakness and asymmetric scapular winging, while bulbar symptoms may manifest later or remain inconspicuous. This can seriously complicate the differential diagnosis, particularly considering the existence of a distal phenotype of FSHD without clear facial weakness.

## 5 Conclusion

The phenotypic variability of VCPDM and the rarity of this pathology are the primary factors contributing to diagnostic challenges. Among the six cases reported here, two exhibited a scapuloperoneal phenotype of MATR3-related distal myopathy, necessitating differential diagnosis with FSHD in one of the cases.

## Data Availability

The datasets for this article are not publicly available due to concerns regarding participant/patient anonymity. Requests to access the datasets should be directed to the corresponding author.
